# Risk prediction of covid-19 related death and hospital admission in adults after covid-19 vaccination: national prospective cohort study

**DOI:** 10.1136/bmj.n2244

**Published:** 2021-09-17

**Authors:** Julia Hippisley-Cox, Carol AC Coupland, Nisha Mehta, Ruth H Keogh, Karla Diaz-Ordaz, Kamlesh Khunti, Ronan A Lyons, Frank Kee, Aziz Sheikh, Shamim Rahman, Jonathan Valabhji, Ewen M Harrison, Peter Sellen, Nazmus Haq, Malcolm G Semple, Peter W M Johnson, Andrew Hayward, Jonathan S Nguyen-Van-Tam

**Affiliations:** 1 Nuffield Department of Primary Health Care Sciences, University of Oxford, Oxford, UK; 2 Division of Primary Care, School of Medicine, University of Nottingham, Nottingham, UK; 3 NHS-X, London, UK; 4 Department of Medical Statistics and Centre for Statistical Methodology, London School of Hygiene and Tropical Medicine, London, UK; 5 Diabetes Research Centre, University of Leicester, Leicester, UK; 6 Population Data Science, Swansea University, Swansea, UK; 7 Queen’s University, Belfast, UK; 8 Usher Institute, University of Edinburgh, Edinburgh, UK; 9 Department of Health and Social Care, England, UK; 10 NHS England and Improvement, London, UK; 11 NIHR Health Protection Research Unit, Institute of Infection, Veterinary and Ecological Sciences, Faculty of Health and Life Sciences, University of Liverpool, Liverpool, UK; 12 UCL Institute of Epidemiology and Health Care, London, UK

## Abstract

**Objectives:**

To derive and validate risk prediction algorithms to estimate the risk of covid-19 related mortality and hospital admission in UK adults after one or two doses of covid-19 vaccination.

**Design:**

Prospective, population based cohort study using the QResearch database linked to data on covid-19 vaccination, SARS-CoV-2 results, hospital admissions, systemic anticancer treatment, radiotherapy, and the national death and cancer registries.

**Settings:**

Adults aged 19-100 years with one or two doses of covid-19 vaccination between 8 December 2020 and 15 June 2021.

**Main outcome measures:**

Primary outcome was covid-19 related death. Secondary outcome was covid-19 related hospital admission. Outcomes were assessed from 14 days after each vaccination dose. Models were fitted in the derivation cohort to derive risk equations using a range of predictor variables. Performance was evaluated in a separate validation cohort of general practices.

**Results:**

Of 6 952 440 vaccinated patients in the derivation cohort, 5 150 310 (74.1%) had two vaccine doses. Of 2031 covid-19 deaths and 1929 covid-19 hospital admissions, 81 deaths (4.0%) and 71 admissions (3.7%) occurred 14 days or more after the second vaccine dose. The risk algorithms included age, sex, ethnic origin, deprivation, body mass index, a range of comorbidities, and SARS-CoV-2 infection rate. Incidence of covid-19 mortality increased with age and deprivation, male sex, and Indian and Pakistani ethnic origin. Cause specific hazard ratios were highest for patients with Down’s syndrome (12.7-fold increase), kidney transplantation (8.1-fold), sickle cell disease (7.7-fold), care home residency (4.1-fold), chemotherapy (4.3-fold), HIV/AIDS (3.3-fold), liver cirrhosis (3.0-fold), neurological conditions (2.6-fold), recent bone marrow transplantation or a solid organ transplantation ever (2.5-fold), dementia (2.2-fold), and Parkinson’s disease (2.2-fold). Other conditions with increased risk (ranging from 1.2-fold to 2.0-fold increases) included chronic kidney disease, blood cancer, epilepsy, chronic obstructive pulmonary disease, coronary heart disease, stroke, atrial fibrillation, heart failure, thromboembolism, peripheral vascular disease, and type 2 diabetes. A similar pattern of associations was seen for covid-19 related hospital admissions. No evidence indicated that associations differed after the second dose, although absolute risks were reduced. The risk algorithm explained 74.1% (95% confidence interval 71.1% to 77.0%) of the variation in time to covid-19 death in the validation cohort. Discrimination was high, with a D statistic of 3.46 (95% confidence interval 3.19 to 3.73) and C statistic of 92.5. Performance was similar after each vaccine dose. In the top 5% of patients with the highest predicted covid-19 mortality risk, sensitivity for identifying covid-19 deaths within 70 days was 78.7%.

**Conclusion:**

This population based risk algorithm performed well showing high levels of discrimination for identifying those patients at highest risk of covid-19 related death and hospital admission after vaccination.

## Introduction

During the first waves of the covid-19 pandemic (March 2020 to August 2020), before the introduction of vaccines, it was essential to be able to identify people at highest risk of adverse outcomes if they were infected with SARS-CoV-2. The QCovid risk assessment tool for predicting risk of covid-19 related death or hospital admission based on individual characteristics was developed,^1^ independently externally validated,^2^ and found to have performed well at identifying those individuals at high risk of severe outcomes from covid-19. The tool was used in England to identify patients at high risk of severe covid-19 outcomes, adding an additional 1.5 million people to the national shielded patient list in February 2021 and, on a UK basis, prioritising them for vaccination (if they had not already been offered the vaccine on account of their age or occupation).^3^

Since then, clinical trials of covid-19 vaccinations have demonstrated safety and efficacy in healthy volunteers^4^ ^5^ ^6^ and have been rolled out to the adult UK population, beginning with the most elderly groups (aged ≥90 years) and those people most at risk. Although vaccines have been found to be highly effective in trials and observational studies, a residual risk of serious covid-19 outcomes (in particular, hospital admission or death) remains after vaccination, despite allowing adequate time for immunity to develop. The risk of a severe outcome in vaccinated groups includes the risk of exposure, the risk of a breakthrough infection if exposed, and the risk of a breakthrough infection becoming severe. However, the relevant risk factors are currently unknown because clinical trials have not included many people in whom vaccine response might be suboptimal (eg, elderly people, people with complex comorbidities (eg, in receipt of solid organ transplants or immunosuppressive treatment for autoimmune disorders), or patients with cancer receiving chemotherapy or radiotherapy^7^).

Therefore, vaccinated individuals at highest risk of consequent severe outcomes such as covid-19 related hospital admission or death need to be identified urgently. A risk stratification tool for the vaccinated population would enable identification of patients to prioritise for targeted, early interventions once these become available—including booster vaccination and preventive treatments such as passive antibody delivery (for either prophylactic or therapeutic use). Risk stratification tools also provide a robust pragmatic mechanism for avoiding unnecessary lifestyle precautions, investigations, and therapeutic interventions for those individuals whose risk is relatively low, but who might perceive it to be much higher.

We developed and validated two new QCovid risk algorithms, based on data from the second pandemic wave in England, to identify those groups at highest risk of severe covid-19 outcomes: QCovid2 (based on unvaccinated patients) and QCovid3 (based on vaccinated patients). Given the nature of the pandemic, the speed of the vaccination programme, the relaxation of lockdown measures, and the urgent need to develop national policy, this work had to be undertaken during the pandemic period and national vaccination programme, and therefore included people who had only one vaccination dose as well as those who were fully vaccinated.

## Methods

### Data sources

We used the QResearch database (version 46) of 12 million patients with personal, clinical, and drug data that have been used for clinical^1^ ^8^ and drug safety research.^9^ ^10^ QResearch is linked to multiple datasets at individual patient level. For this analysis, we used the National Immunisation Database of covid-19 vaccinations to identify data on vaccine date and doses for all people vaccinated in England. For hospital admissions, we used the linked Hospital Episode Statistics dataset supplemented by the more regularly updated Secondary Users Service data. We also obtained and linked the following datasets: national data for mortality; SARS-CoV-2 infection; systemic anticancer treatment; radiotherapy treatment datasets; and national cancer registry data.

### Study design and period for vaccinated cohort

We undertook a prospective cohort study of vaccinated individuals from 8 December 2020 (the earliest vaccination date in England) to 15 June 2021 (the latest date for which data were available at the time of the analysis). We considered outcomes after the first and second vaccination doses. The cohort included people who received one or two doses of a covid-19 vaccine during the study period. Individuals were followed from 14 days after receiving each vaccine dose until they had the outcome of interest, died, or reached the end of the study period. Use of follow-up time after the first dose and after the second dose is described below.

### Inclusion criteria for vaccinated cohort

We included all adults aged 19-100 years who had one or two doses of the ChAdOx1 nCoV-19 (Oxford-AstraZeneca) or BNT162b2 (Pfizer-BioNTech) vaccine during the study period. Both these vaccinations require two doses for full vaccination. People were excluded from the analysis of hospital outcomes if they had a covid-19 associated hospital admission before their start of follow-up (14 days after the first or second dose of vaccination).

### Outcomes for vaccinated cohort

The primary outcome was time to covid-19 related death (either in or out of hospital) as recorded on the death certification, or death within 28 days of a SARS-CoV-2 infection confirmed by reverse transcription polymerase chain reaction (RT-PCR). The secondary outcome was time to hospital admission with covid-19, defined as either confirmed or suspected covid-19 on ICD-10 (international classification of diseases, 10th revision) codes U071 and U072, or new hospital admission associated with a confirmed SARS-CoV-2 infection in the preceding 14 days. These outcome definitions are also used for covid-19 death and hospital admission in the UK.^11^ Both outcomes were assessed from 14 days or more after the first and second doses of vaccination, from which time we considered it was reasonable to expect some immunity.

### Predictor variables for vaccinated cohort

Candidate predictor variables likely to be associated with increased risk of covid-19 death or hospital admission were identified from the original QCovid protocol^12^ and from previous studies.^1^ ^8^ ^13^ The variables were vaccine dose (first or second), age, sex, ethnic origin, Townsend deprivation score (an area level score based on postcode where higher scores indicate higher levels of deprivation^14^), body mass index,^13^ domicile (care home, homeless, neither), chronic kidney disease, chemotherapy in previous 12 months, type 1 or type 2 diabetes (with glycated haemoglobin (HbA_1c_) levels <59 or ≥59 mmol/mol), blood cancer, bone marrow transplantation in past six months, respiratory cancer, radiotherapy in past six months, solid organ transplantation, chronic obstructive pulmonary disease, asthma, rare lung diseases (cystic fibrosis, bronchiectasis, or alveolitis), pulmonary hypertension or pulmonary fibrosis, coronary heart disease, stroke, atrial fibrillation, heart failure, venous thromboembolism, peripheral vascular disease, congenital heart disease, dementia, Parkinson’s disease, epilepsy, rare neurological conditions (motor neurone disease, multiple sclerosis, myasthenia gravis, or Huntington’s chorea), cerebral palsy, osteoporotic fracture, rheumatoid arthritis or systemic lupus erythematosus, liver cirrhosis, bipolar disorder or schizophrenia, inflammatory bowel disease, sickle cell disease, HIV/AIDS, severe combined immunodeficiency, and record of a SARS-CoV-2 positive test result before cohort entry.

To account for changing infection rates during the study period (since the vaccination programme was started during the second pandemic wave in England), we calculated a seven-day moving average of the background rates of positive SARS-CoV-2 tests per 100 000 people, using published English national data.^15^ We linked the rate to the date of cohort entry for each individual (that is, 14 days after each vaccine dose).

We defined predictors using information recorded in primary care electronic health records at the start of follow-up at 14 days after the first dose, except for chemotherapy, radiotherapy, and transplantations, which were based on linked data related to systemic anticancer and radiotherapy treatment, Hospital Episode Statistics data, and Secondary Users Service data. For all predictor variables, we used the most recently available value at the cohort entry date.

### Model development

To maximise the number of events after second dose of vaccine, we used all 1336 practices with linked data available up to 15 June 2021 at the time of model development. We subsequently validated it in the remaining 182 practices once the updated linked data to 15 June 2021 became available a few weeks later.

People entered the cohort at 14 days after their first vaccination dose. We used a landmarking approach^16^ to handle the time dependent dose variable, because some people contributed follow-up time after their second dose as well as after their first dose. For people with only one dose, we followed them up until they had the event of interest, died, or reached the study end. For those with two vaccination doses, we split follow-up time into two periods. Period 1 included the time from 14 days after their first vaccination dose until 14 days after their second dose (therefore, outcomes during the first 14 days after the second dose were attributed to the first dose). Period 2 included time from 14 days after their second dose until they had the event of interest, died, or reached the study end. We fitted all models using combined data from follow-up after the first and second doses, with dose number entered into the model as a predictor.

We developed the risk models using cause specific Cox proportional hazard models to calculate hazard ratios and develop risk scores accounting for the competing risk of death due to other causes. A hazard ratio is a measure of the rate at which a particular outcome happens in one group relative to the rate at which it happens in another group over time. We fitted two cause specific Cox models to derive a risk algorithm for our primary outcome—one for covid-19 deaths and one for deaths due to other causes, censoring patients with the respective competing event. For our secondary outcome, we fitted one model for covid-19 admission and another model for all cause mortality (excluding deaths occurring after a covid-19 admission).

We used second degree fractional polynomials to model non-linear associations for continuous variables including SARS-Cov-2 infection rates, age, body mass index, and Townsend deprivation score.^14^ We fitted the models to the complete cases (that is, with no missing values for predictor variables) to derive the fractional polynomial terms. We used multiple imputation with chained equations to impute missing values for ethnic origin, Townsend score, body mass index, and HbA_1c_. We carried out five imputations and fitted the prediction models in each imputed dataset, and used Rubin’s rules to combine the model parameter estimates across the imputed datasets.^17^

We retained variables in the final models that were significant at the 5% level (taking account of the clustered nature of the data) or when adjusted cause specific hazard ratios for categorical variables were more than 1.1. Clinically similar variables with low numbers of events, such as bone marrow and solid organ transplantation, were combined. We examined interactions between predictor variables and age, as well as interactions between vaccine dose and age, body mass index, ethnic origin, deprivation, and each comorbidity. Furthermore, we derived estimates of the cumulative incidence function for covid-19 mortality accounting for the competing risk of death from other causes by combining estimates obtained from the two cause specific Cox models using an appropriate formula.^18^ The same method was used to derive the cumulative incidence function for covid-19 hospital admission, accounting for competing risk of death. These final algorithms for predicting absolute risk in vaccinated individuals are referred to as QCOVID3.^18^

We developed an additional model restricted to vaccinated patients with a positive SARS-CoV-2 test result after vaccination. This model separately quantified the risk of severe outcomes (mortality and admission) in individuals with a record of infection.

### Model evaluation

We evaluated model performance in the separate validation cohort. We used multiple imputation to replace missing values for ethnic origin, body mass index, and Townsend score; the imputation model used was the same as that used in the derivation cohort. We applied the final risk equations to calculate the risk scores for each outcome accounting for competing risks, and calculated a C index accounting for competing risks using R.^19^ We also calculated R^2^ values and D statistics^20^ although these statistics were only available for the cause specific outcomes.

We assessed model calibration in the validation cohort accounting for competing risks by comparing mean predicted risks with the observed cumulative incidence function by twentieths of predicted risk.^21^ A model is well calibrated if predicted risks closely approximate the observed risks. We calculated each metric in the whole validation cohort, separately for individuals who had received one and two vaccination doses, and in subgroups for age and sex (ethnic groups had too few patients to undertake analyses).

### Risk stratification

We applied the algorithms to the validation cohort to define the centile thresholds based on absolute risk using the prevailing SARS-CoV-2 rate 14 days after the date of each vaccination dose. Sensitivity was calculated as the total cumulative number of patients with a risk score above the risk threshold with a covid-19 death by 70 days divided by the total cumulative number of patients with a covid-19 death by 70 days.

### QCovid2 model in the unvaccinated cohort

We also developed and evaluated two additional models (QCovid2) based on a cohort of unvaccinated people aged 19-100 years and observed between 1 September 2020 and 31 May 2021 but censoring people who were subsequently vaccinated on the date of their first vaccination. Additional variables not included in the original QCovid model were used, such as inflammatory bowel disease and levels of diabetes control according to HbA_1c_ measurements. We also examined separate variables for sickle cell disease, HIV/AIDs, immunodeficiency conditions, and a refined definition of severe mental illness (to determine the contribution of moderate and severe depression). The first model included all unvaccinated patients (restricting to the time before vaccination for those who were subsequently vaccinated). The second model was restricted to unvaccinated patients with a positive SARS-CoV-2 test result, to separately quantify the risk of SARS-CoV-2 infection from the risk of severe outcomes (covid-19 mortality and admission) in those people with a positive test result. These final algorithms for predicting absolute risk in unvaccinated individuals are referred to as QCovid2.

### Reporting

Stata (version 17) and R were used for analyses. The study adhered to the TRIPOD (transparent reporting of a multivariable prediction model for individual prognosis or diagnosis) statement for reporting.^22^

### Patient and public involvement

Patients were involved in framing the research question, identifying predictors, and in developing plans for design and implementation of the study. 

## Results

### Characteristics of the vaccinated cohort

Table 1 shows the characteristics of the 6 952 440 vaccinated patients in the derivation cohort, of whom 4 026 592 (57.9%) had the Oxford-AstraZeneca vaccine and 2 925 848 (42.1%) had the Pfizer-BioNTech vaccine. Overall, the mean age was 52 years (standard deviation 17.7), 3 321 247 (47.8%) were men, and 5 150 310 (74.1%) had two vaccine doses. The median follow-up time was 72 days (interquartile range 59-77) after the first dose and 35 (18-53) days after the second dose. Of 2031 covid-19 related deaths and 1929 covid-19 related hospital admissions, 81 deaths and 71 admissions occurred 14 days or more after the second vaccine dose. Of the 1929 patients in hospital, 446 (23.1%) subsequently died. Supplementary table 1 shows corresponding results for the 626 656 vaccinated patients in the validation cohort, of whom 174 had a covid-19 death and 179 had a covid-19 hospital admission. Of these, 10 deaths and seven admissions occurred 14 days or more after the second vaccine dose.

**Table 1:** Personal and medical characteristics for the derivation cohort and covid-19 related death or hospital admission 14 days or more after vaccination. Data are number (%) unless stated otherwise

Characteristics	Total (n=6 952 440)	Covid-19 deaths (n=2031)	Covid-19 admissions (n=1929)
Sex:			
Women	3 631 193 (52.23)	981 (48.30)	983 (50.96)
Men	3 321 247 (47.77)	1050 (51.70)	946 (49.04)
Mean age (SD)	52.46 (17.73)	84.48 (9.15)	77.36 (14.84)
Mean Townsend deprivation score (SD)	−0.17 (2.98)	−0.25 (2.76)	−0.05 (2.92)
Mean body mass index (SD)	27.30 (5.64)	26.04 (5.89)	27.90 (6.18)
Mean background SARS-CoV-2 daily infection rate per 100 000 population (SD)	21.34 (22.80)	60.05 (21.06)	52.93 (22.38)
No of patients with SARS-Co-2 positive test result before vaccination	414 163 (5.96)	147 (7.24)	78 (4.04)
Age (years):			
<30	771 125 (11.09)	—	19 (0.98)
30-39	1 105 120 (15.90)	—	43 (2.23)
40-49	1 218 902 (17.53)	9 (0.44)	71 (3.68)
50-59	1 402 707 (20.18)	38 (1.87)	121 (6.27)
60-69	1 090 778 (15.69)	81 (3.99)	160 (8.29)
70-79	860 179 (12.37)	327 (16.10)	377 (19.54)
80-89	414 752 (5.97)	960 (47.27)	830 (43.03)
≥90	88 877 (1.28)	614 (30.23)	308 (15.97)
Covid-19 vaccination:			
1 dose	1 802 130 (25.92)	1947 (95.86)	1858 (96.32)
2 doses	5 150 310 (74.08)	81 (4.14)	71 (3.68)
Ethnic origin:			
White	4 781 050 (68.77)	1512 (74.45)	1466 (76.00)
Indian	202 528 (2.91)	44 (2.17)	51 (2.64)
Pakistani	111 873 (1.61)	27 (1.33)	46 (2.38)
Bangladeshi	81 197 (1.17)	8 (0.39)	14 (0.73)
Other Asian	117 061 (1.68)	13 (0.64)	22 (1.14)
Caribbean	48 486 (0.70)	15 (0.74)	13 (0.67)
Black African	113 663 (1.63)	4 (0.20)	11 (0.57)
Chinese	41 595 (0.60)	—	—
Other	187 576 (2.70)	15 (0.74)	20 (1.04)
Chronic kidney disease:			
None	6 597 783 (94.90)	1231 (60.61)	1290 (66.87)
Stage 3	319 898 (4.60)	662 (32.59)	531 (27.53)
Stage 4	17 914 (0.26)	85 (4.19)	56 (2.90)
Stage 5 only	10 098 (0.15)	45 (2.22)	27 (1.40)
Stage 5 with dialysis	2182 (0.03)	—	10 (0.52)
Stage 5 with transplant	4565 (0.07)	5 (0.25)	15 (0.78)
Chemotherapy:			
None in past 12 months	6 911 085 (99.41)	1978 (97.39)	1891 (98.03)
Group A	14 518 (0.21)	9 (0.44)	12 (0.62)
Group B	25 087 (0.36)	42 (2.07)	25 (1.30)
Group C	1750 (0.03)	—	—
Type 1 diabetes:			
No type 1 diabetes	6 911 191 (99.41)	2023 (99.61)	1919 (99.48)
HbA_1c_ ≤59 mmol/mmol (≤7.5%)	13 536 (0.19)	—	—
HbA_1c_ >59 mmol/mol (>7.5%)	27 276 (0.39)	5 (0.25)	9 (0.47)
HbA_1c_ not recorded	437 (0.01)	—	—
Type 2 diabetes:			
No type 2 diabetes	6 375 340 (91.70)	1486 (73.17)	1385 (71.80)
HbA_1c_ ≤59 mmol/mol (≤7.5%)	370 653 (5.33)	382 (18.81)	347 (17.99)
HbA_1c_ >59 mmol/mol (>7.5%)	203 998 (2.93)	159 (7.83)	196 (10.16)
HbA_1c_ not recorded	2449 (0.04)	—	—
Other pre-existing health conditions:			
Blood cancer	46 748 (0.67)	72 (3.55)	67 (3.47)
Bone marrow transplantation in past 6 months or solid organ transplantation ever	1979 (0.03)	—	7 (0.36)
Respiratory cancer	17 401 (0.25)	29 (1.43)	19 (0.98)
Radiotherapy in past 6 months	12 011 (0.17)	19 (0.94)	16 (0.83)
Down’s syndrome	3963 (0.06)	—	—
Chronic obstructive pulmonary disease	199 780 (2.87)	278 (13.69)	216 (11.20)
Coronary heart disease	318 851 (4.59)	530 (26.10)	456 (23.64)
Stroke	193 710 (2.79)	407 (20.04)	282 (14.62)
Atrial fibrillation	222 783 (3.20)	479 (23.58)	399 (20.68)
Heart failure	105 427 (1.52)	308 (15.16)	241 (12.49)
Venous thromboembolism	158 464 (2.28)	216 (10.64)	140 (7.26)
Peripheral vascular disease	63 553 (0.91)	131 (6.45)	97 (5.03)
Dementia	81 320 (1.17)	631 (31.07)	305 (15.81)
Parkinson’s disease	22 489 (0.32)	84 (4.14)	38 (1.97)
Epilepsy	109 204 (1.57)	49 (2.41)	61 (3.16)
Rare neurological conditions	27 312 (0.39)	20 (0.98)	18 (0.93)
Liver cirrhosis	17 457 (0.25)	27 (1.33)	19 (0.98)
Sickle cell disease	2073 (0.03)	—	—
HIV/AIDS	15 218 (0.22)	—	—
Severe combined immunodeficiency	3853 (0.06)	—	—

SD=standard deviation; HbA_1c_=glycated haemogoblin. Chemotherapy groups are defined in supplementary box A of reference 1.

### QCovid3: associations of outcomes with predictor variables

**Fig 1:**
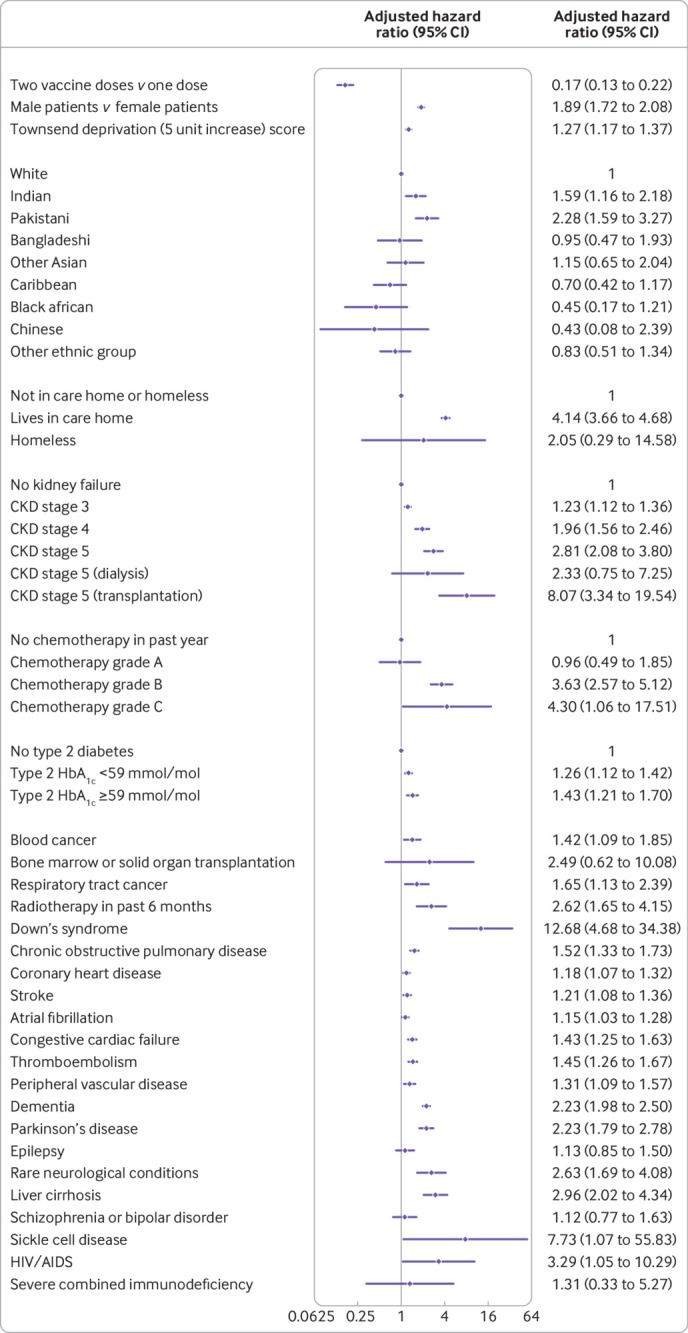
Use of QCovid3 model showing adjusted cause specific hazard ratios for covid-19 death after vaccination, mutually adjusted and adjusted for fractional polynomial terms for age, body mass index, vaccination dose, and background infection rate at time of vaccination. CKD=chronic kidney disease; HbA_1c_=glycated haemogoblin

**Fig 2:**
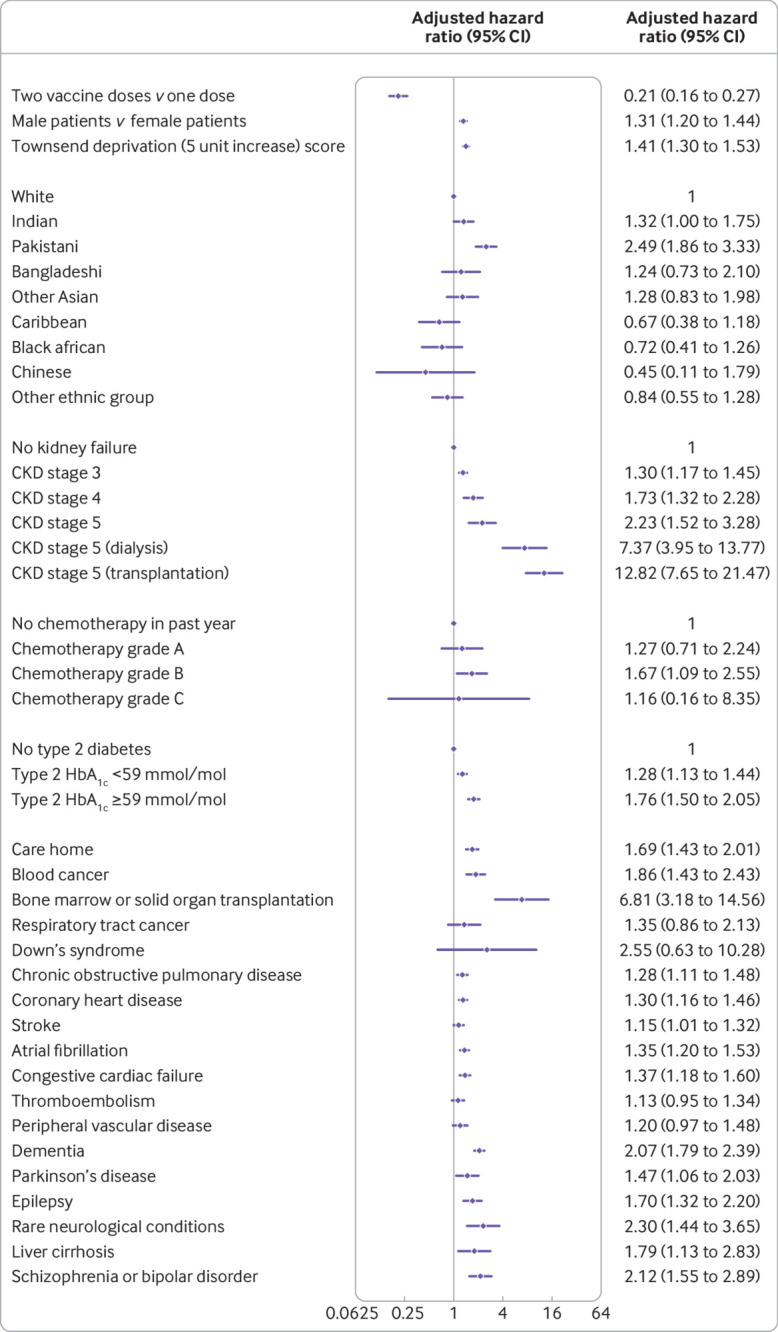
Use of QCovid3 model showing adjusted cause specific hazard ratios for covid-19 hospital admission after vaccination, mutually adjusted and adjusted for fractional polynomial terms for age, body mass index, vaccination dose, and background infection rate at time of vaccination. CKD=chronic kidney disease; HbA_1c_=glycated haemogoblin

The final risk algorithms for covid-19 mortality included age, sex, ethnic origin, Townsend deprivation, body mass index, a range of comorbidities, and SARS-CoV-2 infection rate. We did not find any evidence of interactions between the dose variable and other predictors (although we did find small numbers for some pre-existing health conditions). Figure 1 and figure 2 show the adjusted hazard ratios and 95% confidence intervals for predictor variables included in the final cause specific models for covid-19 related deaths and hospital admissions. Supplementary figures 1-3 show the adjusted hazard ratios for the fractional polynomial terms for age, body mass index, and background SARS-CoV-2 infection rate, respectively. Hazard ratios increased with increasing age, Townsend deprivation, and background rates of SARS-CoV-2 infection. We saw a J shaped association between body mass index and rates of both hospital admission and mortality outcomes. Supplementary figures 4 and 5 show the adjusted hazard ratios for the competing events of non-covid-19 death and all cause deaths.

Covid-19 mortality incidence increased with age and deprivation, male sex, and Indian and Pakistani ethnic origin. Hazard ratios were highest for those with Down’s syndrome (12.7-fold increase), kidney transplantation (8.1-fold), sickle cell disease (7.7-fold), care home residency (4.1-fold), group B (3.6-fold) and group C chemotherapy (4.3-fold), recent bone marrow transplantation or a solid organ transplantation ever (2.5-fold), HIV/AIDS (3.3-fold), dementia (2.2-fold), Parkinson’s disease (2.2-fold), neurological conditions (2.6-fold), and liver cirrhosis (3.0-fold). Other conditions associated with increased covid-19 mortality included chronic kidney disease, blood cancer, epilepsy, chronic obstructive pulmonary disease, coronary heart disease, stroke, atrial fibrillation, heart failure, thromboembolism, peripheral vascular disease, and type 2 diabetes (with highest risks among those with HbA_1c_ ≥59 mmol/mol (>7.5%)). The adjusted hazard ratio for covid-19 related death was 0.17 (95% confidence interval 0.13 to 0.22) after the second vaccine dose (plus 14 days) compared with after the first vaccine dose (plus 14 days).

We found similar patterns of associations between predictors and the cause specific hazard for covid-19 admission (fig 2) except for conditions with too few events for analysis (that is, sickle cell disease, severe combined immunodeficiency, and HIV/AIDS). Similarly, the adjusted hazard ratio of covid-19 related hospital admission was 0.21 (95% confidence interval 0.16 to 0.27) after the second dose compared with after the first dose.

Supplementary figure 6 shows the corresponding results for risk of covid-19 death among the subgroup of patients with a SARS-CoV-2 positive test result. The associations for each factor in the restricted model were similar to those of the main QCovid3 model apart from ethnic origin (for which no significant associations were seen) and conditions with too few events for analysis (sickle cell disease, severe combined immunodeficiency, HIV/AIDS). All associations with pre-existing health conditions reported are conditional on the other predictors in the model and do not necessarily have a causal interpretation.

### QCovid3 model evaluation of performance

Table 2 shows the performance of the risk equations in the validation cohort. The QCovid3 algorithm for covid-19 related death explained 74.1% (95% confidence interval 71.1% to 77.0%) of the variation in time to covid-19 death, the Royston’s D statistic was 3.46 (3.19 to 3.73) and the Harrell’s C statistic was 92.5. Results were similar in men and women. Corresponding results restricted to the first vaccine dose were 71.3% (67.9% to 74.7%), 3.23 (2.96 to 3.50), and 93.6. The results restricting to the validation cohort after the second vaccine dose were similar but with wider confidence intervals owing to smaller numbers. The values for the R^2^, D, and C statistics were similar for the hospital admission equation. Supplementary table 2 shows the corresponding results for covid-19 death and hospital admission by age band where performance tended to be lower in the higher age bands.

**Table 2:** Performance of QCovid3 risk model in the validation cohort for covid-19 related death and hospital admission

	Covid-19 death	Covid-19 admission
**Overall**		
Harrell’s C statistic	92.5	85.3
R^2^	74.1 (71.1 to 77)	65.7 (61.8 to 69.6)
Royston’s D statistic	3.46 (3.19 to 3.73)	2.83 (2.59 to 3.08)
**Women**		
Harrell’s C statistic	94.4	86.8
R^2^	75.4 (71.6 to 79.3)	66.4 (60.9 to 71.9)
Royston’s D statistic	3.59 (3.22 to 3.96)	2.88 (2.52 to 3.23)
**Men**		
Harrell’s C statistic	90.4	83.6
R^2^	72.7 (68.5 to 76.9)	64.9 (59.5 to 70.4)
Royston’s D statistic	3.34 (2.99 to 3.7)	2.79 (2.45 to 3.12)
**One dose of vaccine only**		
Harrell’s C statistic	93.6	85.5
R^2^	71.3 (67.9 to 74.7)	60 (55.2 to 64.7)
Royston’s D statistic	3.23 (2.96 to 3.5)	2.5 (2.26 to 2.75)
**Two doses of vaccine**		
Harrell’s C statistic	81.7	79.3
R^2^	70 (54.5 to 85.6)	72.1 (57.3 to 87)
Royston’s D statistic	3.13 (1.97 to 4.29)	3.29 (2.08 to 4.51)

Harrell’s C statistic=time dependent area under the curve accounting for competing risks; confidence intervals could not be obtained.

**Fig 3:**
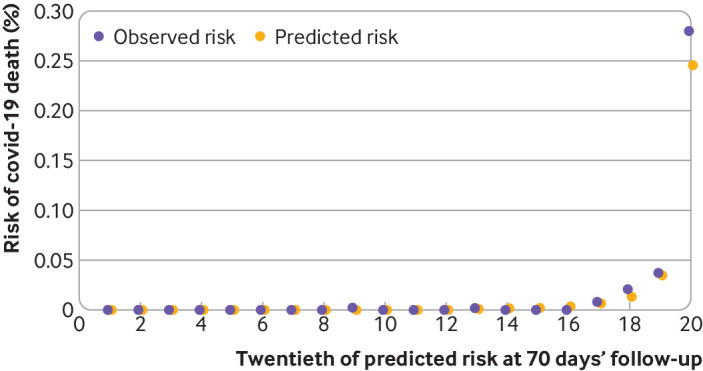
Calibration of the QCovid3 risk model to predict covid-19 related death after vaccination. Data source: QResearch England, 8 December 2020 to 15 June 2021, https://www.qresearch.org/

**Fig 4:**
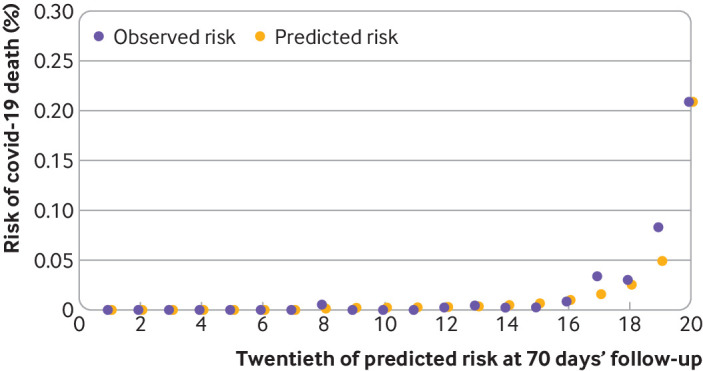
Calibration of the QCovid3 risk model to predict covid-19 related hospital admission after vaccination. Data source: QResearch England, 8 December 2020 to 15 June 2021, https://www.qresearch.org/

Figure 3 shows the calibration plot for covid-19 related deaths and figure 4 shows the corresponding results for covid-19 related hospital admission, both accounting for competing risks. Model calibration was assessed by comparing mean predicted risks with the observed cumulative incidence function by twentieths of predicted risk^21^; a model is well calibrated if predicted risks closely approximate the observed risks. These plots showed reasonable correspondence between observed and predicted cumulative incidences at 70 days of follow-up. However, numbers of events were small in several groups, and in the higher twentieths we saw slight under-prediction for covid-19 death (fig 3) and in twentieths 17-19 for the admissions outcome (fig 4). For example, in the top twentieth of predicted risks for covid-19 death, the observed cumulative incidence was 0.28% over 70 days (95% confidence interval 0.24% to 0.33%) and the mean predicted risk was 0.25%.

### QCovid3 risk stratification

Table 3 shows the percentage of covid-19 related deaths identified by the QCovid3 mortality equation at different thresholds based on centiles of predicted absolute risk in the validation cohort, using the background SARS-CoV-2 infection rate associated with 14 days after vaccination. For example, it shows that 78.7% of deaths occurred in individuals in the top 5% for predicted absolute risk of covid-19 death (predicted absolute risks at 70 days above 0.06%). Individuals in the top 20% for predicted absolute risk of death accounted for 98.9% of deaths. Table 4 summarises the characteristics of individuals at the highest predicted absolute risk of covid-19 death (top 5%). Box 1 lists clinical examples of patients and their predicted covid-19 risks (https://bmjSept2021.qcovid.org).

**Table 3:** Sensitivity for covid-19 related death at 70 days’ follow-up in the validation cohort (consisting of 626 656 vaccinated patients with 174 covid-19 related deaths at different QCovid3 thresholds of absolute risk)

Centile threshold of predicted absolute risk	Threshold predicted absolute risk (%) at 70 days	Total No of cumulative deaths	Cumulative proportion (%) of deaths based on absolute risk (sensitivity)*
Top 5%	0.061	137	78.74
Top 10%	0.020	157	90.23
Top 15%	0.009	167	95.98
Top 20%	0.005	172	98.85
Top 25%	0.003	172	98.85
Top 30%	0.003	172	98.85

* Sensitivity calculated as the total cumulative number of patients with a risk score above the risk threshold with a covid-19 death at 70 days divided by the total cumulative number of patients with a covid-19 death at 70 days.

**Table 4:** Characteristics of patients at highest risk of covid-19 related death (top 5%) from 14 days after covid-19 vaccination in the validation cohort using QCovid3 model. Data are numbers (percentages) unless stated otherwise

Characteristics	Patients in top 5% highest risk of covid-19 death (n=28 751)	Patients not in top 5% highest risk of covid-19 death (n=546 281)
14 days after vaccination:		
Covid-19 related death	115 (0.40)	49 (0.01)
Covid-19 related hospital admission	86 (0.30)	86 (0.02)
Sex:		
Women	12 627 (43.92)	290 694 (53.21)
Men	16 124 (56.08)	255 587 (46.79)
Mean age (SD)	85.48 (5.65)	53.39 (15.52)
Mean Townsend deprivation score (SD)	−0.21 (2.94)	0.01 (3.05)
Mean body mass index (SD)	27.23 (5.50)	27.76 (5.68)
Ethnic origin:		
White	26 508 (92.20)	463 902 (84.92)
Indian	848 (2.95)	18 339 (3.36)
Pakistani	402 (1.40)	8441 (1.55)
Bangladeshi	53 (0.18)	4063 (0.74)
Other Asian	158 (0.55)	9178 (1.68)
Caribbean	434 (1.51)	6275 (1.15)
Black African	52 (0.18)	14 708 (2.69)
Chinese	26 (0.09)	3590 (0.66)
Other	270 (0.94)	17 785 (3.26)
Chronic kidney disease:		
None	18 259 (63.51)	528 091 (96.67)
Stage 3	9237 (32.13)	16 640 (3.05)
Stage 4	827 (2.88)	609 (0.11)
Stage 5 only	309 (1.07)	387 (0.07)
Stage 5 with dialysis	52 (0.18)	161 (0.03)
Stage 5 with transplantation	67 (0.23)	393 (0.07)
Chemotherapy:		
None in past 12 months	28 035 (97.51)	543 184 (99.43)
Group A	144 (0.50)	1242 (0.23)
Group B	536 (1.86)	1712 (0.31)
Group C	36 (0.13)	143 (0.03)
Type 2 diabetes:		
No type 2 diabetes	20 607 (71.67)	501 199 (91.75)
HbA_1c_ ≤59 mmol/mol (≤7.5%)	5794 (20.15)	29 176 (5.34)
HbA_1c_ >59 mmol/mol (>7.5%)	2350 (8.17)	15 906 (2.91)
Other pre-existing health conditions:		
Blood cancer	838 (2.91)	2824 (0.52)
Bone marrow or solid organ transplant	21 (0.07)	133 (0.02)
Respiratory cancer	377 (1.31)	1034 (0.19)
Recent radiotherapy	219 (0.76)	646 (0.12)
Down’s syndrome	39 (0.14)	319 (0.06)
Chronic obstructive pulmonary disease	4018 (13.98)	15 265 (2.79)
Coronary heart disease	7489 (26.05)	20 457 (3.74)
Stroke	4627 (16.09)	11 039 (2.02)
Atrial fibrillation	5986 (20.82)	11 266 (2.06)
Heart failure	3316 (11.53)	4694 (0.86)
Venous thromboembolism	2545 (8.85)	10 322 (1.89)
Dementia	3698 (12.86)	1407 (0.26)
Parkinson’s disease	635 (2.21)	987 (0.18)
Epilepsy	549 (1.91)	9286 (1.70)
Rare neurological conditions	220 (0.77)	2131 (0.39)
Liver cirrhosis	253 (0.88)	1188 (0.22)
Sickle cell disease	12 (0.04)	196 (0.04)
HIV/AIDS	36 (0.13)	1667 (0.31)
Severe combined immunodeficiency	22 (0.08)	280 (0.05)

SD=standard deviation; HbA_1c_=glycated haemogoblin. Chemotherapy groups are defined in supplementary box A of reference 1.

Box 1 **Clinical examples of patients and their predicted covid-19 risks over a 70 day period, based on QCovid3 risk algorithms (https://bmjSept2021.qcovid.org)**

**Example 1**

72 year old white man with a first vaccine dose, atrial fibrillation, and body mass index of 30 (background daily infection rate of 22 positive reverse transcription polymerase chain reaction (RT-PCR) test results per 100 000 people) would have:

- 0.04% risk of covid-19 related hospital admission over a 70 day period
- 0.02% risk of covid-19 death over a 70 day period
- 5.15% risk of covid-19 related death after a SARS-CoV-2 positive test result.

**Example 2**

62 year old Pakistani woman with two vaccine doses, chronic kidney disease stage 5 with transplantation, and body mass index of 24 (background daily infection rate of 20 positive RT-PCR test results per 100 000 people) would have:

- 0.04% risk of covid-19 related hospital admission over a 70 day period
- 0.003% risk of covid-19 death over a 70 day period
- 0.10% risk of covid-19 related death after a SARS-CoV-2 positive test result.

**Example 3**

60 year old white man with a first vaccine dose, stroke, epilepsy, well controlled type 2 diabetes, Down’s syndrome, and body mass index of 41 (background daily infection rate of 60 positive RT-PCR test results per 100 000 people) would have:

- 0.56% risk of covid-19 related hospital admission over a 70 day period
- 0.46% risk of covid-19 death over a 70 day period
- 24.3% risk of covid-19 related death after a SARS-CoV-2 positive test result.

**Example 4**

67 year-old Caribbean woman with a first vaccine dose, liver cirrhosis, and body mass index of 41 (background daily infection rate of 40 positive RT-PCR test results per 100 000 people) would have:

- 0.08% risk of covid-19 related hospital admission over a 70 day period
- 0.04% risk of covid-19 death over a 70 day period
- 7.29% risk of covid-19 related death after a SARS-CoV-2 positive test result.

### QCovid2 model in unvaccinated patients

Supplementary figure 7 shows the adjusted hazard ratios for risk of covid-19 death for men and women in the comparison cohort of unvaccinated individuals using the QCovid2 model. Supplementary figure 8 shows the corresponding results for covid-19 hospital admission. These models are updated versions of the original QCovid model from our earlier publication.^1^ The new models include additional variables for inflammatory bowel disease, levels of diabetes control (according to measured HbA_1c_ values), separate variables for sickle cell disease, HIV/AIDS, and severe combined immunodeficiency, and a refined definition of severe mental illness (which now includes only schizophrenia or bipolar disease but does not include moderate and severe depression).

The hazard ratios for QCovid2 were generally similar in magnitude and direction for the subset of variables included in our main model (QCovid3). However, some additional variables included in the QCovid2 model did not reach statistical significance or resulted in hazard ratios lower than 1.1, and hence were not included in the main QCovid3 model. These variables were type 1 diabetes, asthma, rare lung conditions, pulmonary fibrosis or pulmonary hypertension, congenital heart disease, cerebral palsy, inflammatory bowel disease, and severe mental illness (schizophrenia or bipolar disorder).

Supplementary table 3 shows similar performance statistics for discrimination and explained variation for the main QCovid2 model when compared with the original QCovid model evaluated in the validation cohort. Supplementary figures 9 and 10 show adjusted hazard ratios from a similar analysis for risk of covid-19 death and hospital admission in unvaccinated patients but restricted to individuals with a positive SARS-CoV-2 test result.

## Discussion

### Principal findings

We have identified a range of important clinical risk factors for severe covid-19 outcomes in people in the UK, 14 days or more after covid-19 vaccination (first or second dose) when some immunity is expected to have developed. We have used national linked datasets from general practice, national immunisation and SARS-CoV-2 testing, death registry, and hospital episode data for a population representative sample of more than 6.9 million adults. Risk ratios were highest for people with Down’s syndrome, kidney transplantation, sickle cell disease, care home residency, chemotherapy, recent bone marrow transplantation or solid organ transplantation ever, HIV/AIDS, dementia, Parkinson’s disease, neurological conditions, and liver cirrhosis. We also developed and evaluated novel clinical risk prediction models to estimate the absolute risks of covid-19 related hospital admission and mortality in the general population of vaccinated people as well as in the subset of people with positive SARS-CoV-2 test results. The risk models showed high levels of discrimination (C statistics ≥0.88 for the primary outcome, covid-19 death) and good calibration.

For many of the predictors included in the original QCovid model (contributing to risk prediction in an unvaccinated population during wave 1), the magnitude of the relative risks is broadly comparable in both QCovid2 (risk prediction in an unvaccinated population, wave 2) and QCovid3 (risk prediction in a vaccinated population, wave 2). Although these associations cannot be given a causal interpretation, individual characteristics such as age,^23^ obesity, pre-existing medical conditions, and socioeconomic disadvantage^24^ are known to affect immune competence^25^ and, at least for certain diseases, affect the response to some vaccines^26^ ^27^ ^28^ ^29^ ^30^ or to immunosuppressive drugs.^7^ ^31^ The associations with Down’s syndrome in all the models are likely to reflect increased susceptibility to infection and genetic predisposition.^8^ Compared with the white ethnic group, the Pakistani and Indian groups had up to twofold increased hazards of covid-19 death and hospital admission after vaccination in the full QCovid3 model. These ethnic disparities in covid-19 outcomes could represent residual differential exposure (eg, linked to behaviour, lifestyle, household size, and occupation) more than differential susceptibility mechanisms,^32^ although we also acknowledge that being vaccinated could change behaviour (and exposure) in some groups more than in others.

These risk models can be deployed in several health and care settings, either during the current phase of the pandemic or in subsequent waves of infection (with recalibration as required); however, absolute risk for individuals will always depend on disease prevalence and personal exposures. Uses of QCovid3 could include supporting targeted recruitment for clinical trials, prioritisation of vaccine boosters, future preventive treatments such as prophylactic passive monoclonal antibody protection, shielding, and discussions between individuals and clinicians on workplace or health risk mitigation (eg, through improved glycaemic control, weight reduction,^33^or general risk avoidance behaviours). Our QCovid3 model provides absolute risks conditional on patient characteristics, including whether they have received one or two doses of a covid-19 vaccine, and on the underlying prevailing infection levels. It also enables individuals to be ranked in terms of their risk. The deployment of drug and non-drug interventions to protect individuals with residual vulnerability after vaccination needs to be considered in the context of absolute risks of severe outcomes at the time of making predictions. Absolute risks are related to both the prevalence of SARS-CoV-2 infection in the population and the likelihood of SARS-CoV-2 exposure in a vaccinated adult population. Although these algorithms have been designed to inform UK health policy and interventions to manage covid-19 related risks, they also have international potential, subject to local validation. Previous similar risk prediction models have been validated internationally and shown to have good performance outside of the UK.^34^ ^35^

### Strengths and limitations of this study

Our study has some major strengths but also some important limitations, which include specific issues related to covid-19 along with factors similar to those for several other widely used clinical risk prediction algorithms developed using the QResearch database.^36^ ^37^ ^38^ Key strengths included the use of large, validated, representative, population based contemporaneous data sources that have been used to develop other widely used risk prediction tools^36^ ^37^; the wealth of candidate risk predictors; the prospective recording of outcomes and their ascertainment using linkage of multiple national databases; lack of selection, recall, and respondent biases; and robust statistical analysis. We have used non-linear terms to model body mass index, age, and background SARS-CoV-2 infection rates. The inclusion of infection rates is a substantial improvement compared with the original QCovid model, because it enables risks to be updated according to the background infection, which is important given the nature of pandemic waves. Our analysis has been able to separately quantify the risk of severe outcomes among those people with a positive SARS-CoV-2 test result, which was not possible in the original QCovid model^1^ owing to a lack of testing data. Therefore, the analysis could be used at the point of testing to identify those who might benefit from additional interventions such as monoclonal antibodies once these treatments become available.

Limitations included a relatively short duration of follow-up, a partially vaccinated population, and small numbers of events in some subgroups—which are inevitable consequences of undertaking an analysis during rapid deployment of a national vaccination programme. Our analysis incorporated information on whether an individual had received one or two vaccination doses in our prediction models. We saw relatively few deaths in individuals who had received the second dose of the vaccine (4% of all covid-19 related deaths); therefore, most information about associations between predictors and mortality came from individuals who had received only one dose. Results from individuals who have received the second vaccination dose are likely to be most relevant for UK adults as full vaccine coverage increases. Although we examined for interactions, our study might have lacked power to detect whether certain associations differed according to whether one or two doses had been received. Our models also incorporated information on prevailing positive SARS-CoV-2 infection rates, as a proxy for a person’s risk of covid-19 infection at the start of the follow-up period. 

We did not include information on different variants that emerged during the study period owing to incomplete data, particularly in those patients admitted to hospital.^39^ While we accounted for many risk factors for covid-19 mortality, some risks could remain, such as those conferred by rare medical conditions or other factors associated with exposure (eg, occupation) that are poorly recorded in general practice or hospital records and that might be being proxied to some extent by the covariates included. We did not distinguish vaccination type because this study was not designed to compare vaccine effectiveness. Younger patients without underlying health conditions had limited data, because the vaccination programme in England prioritised elderly patients and those people at highest risk. Furthermore, those patients who had two vaccines early in the pandemic were judged to be at highest risk of infection or severe outcomes. 

Although we have reported a validation using practices from QResearch, these practices were separate to those used to develop the model. Previously we have used this approach to develop and validate other widely used prediction models. When these models have been validated on different clinical computer systems, the results have been similar.^40^ ^41^ ^42^ Work is already underway to evaluate the new models in external datasets (such as the English national dataset hosted by the Office for National Statistics) including data from other general practice computer systems that have not been used to derive the algorithm. These data offer a fully independent dataset including data from general practice computer systems not included in the derivation of the dataset. They also offer a larger sample size for validation because clinical and demographic subgroups will have more events. Work is also underway to consider integration of this new algorithm within NHS clinical software systems.

### Policy implications and conclusions

This study presents a robust risk prediction model (QCovid3) that can be used to stratify risk populations to identify those who are at highest risk of severe covid-19 outcomes despite covid-19 vaccination, and who might therefore benefit from further interventions to reduce risk or boost immunity once these become available. The model can be used in conjunction with QCovid2, which updates and replaces the original algorithm (QCovid1) and is designed for use in unvaccinated patients. We anticipate that these algorithms will be updated as the vaccination programme progresses and is extended to younger age groups, as understanding of covid-19 increases, as more post-vaccination follow-up data become available, as new variants of concern emerge, and in response to new policy interventions.

**What is already known on this topic**

The original QCovid tool for predicting risk of covid-19 related death or hospital admission based on individual characteristics was used in England to identify patients at high risk of severe covid-19 outcomes
Identification of these high risk patients added an additional 1.5 million people to the national shielded patient list in February 2021
On a UK basis, these patients would be prioritised for vaccination, if they had not already been offered the vaccine on account of their age or occupation

**What this study adds**

Commissioned by the Chief Medical Officer for England on behalf of the UK government, two new risk prediction algorithms have been derived and validated to estimate the risk of covid-19 related mortality and hospital admission in UK adults, 14 days or more after vaccination when some immunity is expected to have developed
Several clinical risk factors for severe covid-19 outcomes despite vaccination have been identified: Down’s syndrome, kidney transplantation, sickle cell disease, care home residency, chemotherapy, recent bone marrow transplantation or a solid organ transplantation ever, HIV/AIDS, dementia, Parkinson’s disease, neurological conditions, and liver cirrhosis
The QCovid3 risk algorithms (https://bmjSept2021.qcovid.org) showed high levels of discrimination for identifying adults at highest risk of covid-19 related death and hospital admission after vaccination; these risk stratification tools can help support public health policy and prioritise patients for targeted, early interventions

## Data Availability

To guarantee the confidentiality of personal and health information, only the authors have had access to the data during the study in accordance with the relevant licence agreements. Access to the QResearch data is according to the information on the QResearch website (https://www.qresearch.org/). The full model, model coefficients, functional form and cumulative incidence function are published on the qcovid.org website.

## Web extra

Extra material supplied by authors

Web appendix: Supplementary material

## Notes

Contributors: Study conceptualisation was led by JH-C, CACC, and JSN-V-T. JH-C specified the data, organised data approvals, and data linkage. JH-C, CACC, RHK, and KD-O designed the statistical analysis plan. JH-C and CACC undertook the analyses. JH-C wrote the first draft of the paper and developed the software for the web calculator. The Office of the Chief Medical Officer contributed to the development of the study question and facilitated access to relevant national datasets, contributed to interpretation of data, and contributed to the drafting of the report. All authors contributed to the interpretation of the results and revision of the manuscript and approved the final version of the manuscript. JH-C had full access to all data in the study and takes responsibility for the integrity of the data and the accuracy of the data analysis. JH-C is the guarantor for the study. The corresponding author attests that all listed authors meet authorship criteria and that no others meeting the criteria have been omitted.

The lead author affirms that the manuscript is an honest, accurate, and transparent account of the study being reported; that no important aspects of the study have been omitted; and that any discrepancies from the study as planned (and, if relevant, registered) have been explained.

Dissemination to participants and related patient and public communities: Patients will be invited to advise on disseminating the results.

Provenance and peer review: Not commissioned; externally peer reviewed.
