# Supplementary material for: Risk prediction of covid-19 related death and hospital admission in adults after covid-19 vaccination: national prospective cohort study
Source: BMJ. 2021 Sep 17;374:n2244. doi: 10.1136/bmj.n2244 (PMC8446717; doi:10.1136/bmj.n2244)

Predicting risk of death and hospitalisation from COVID-19 in adults following COVID-19 vaccination: national prospective cohort study.

Supplementary Table 1: Characteristics of 626,656 patients in the validation cohort and those with COVID-19 death and COVID-19 admission 14+ days following COVID-19 vaccination

	Total	COVID-19 deaths	COVID-19 admissions
Total	626,656	174	179
Women	327,309 (52.23)	85 (48.85)	82 (45.81)
Men	299347 (47.77)	89 (51.15)	97 (54.19)
Mean age (SD)	52.92 (17.61)	83.75 (9.46)	76.29 (16.23)
Mean Townsend deprivation (SD)	0.07 (3.05)	-0.25 (2.88)	0.08 (2.98)
Mean BMI(SD)	27.59 (5.71)	27.60 (5.80)	29.11 (5.85)
Mean SARS-CoV-2 daily infection rate	21.58 (22.77)	57.21 (21.42)	51.03 (22.75)
< 30 years	64727 (10.33)	*	5 (2.79)
30-39 years	98374 (15.70)	*	5 (2.79)
40-49 years	107436 (17.14)	*	*
50-59 years	128268 (20.47)	*	13 (7.26)
60-69 years	101729 (16.23)	12 (6.90)	17 (9.50)
70-79 years	80604 (12.86)	28 (16.09)	41 (22.91)
80-89 years	37760 (6.03)	81 (46.55)	65 (36.31)
90+ years	7758 (1.24)	49 (28.16)	31 (17.32)
COVID-19 vaccine 1 dose only	152427 (24.32)	164 (94.3)	172 (96.1)
COVID-19 vaccine 2 doses	474229 (75.68)	10 (5.8)	7 (3.9)
White	420742 (67.14)	133 (76.44)	131 (73.18)
Indian	17471 (2.79)	*	7 (3.91)
Pakistani	8382 (1.34)	*	*
Bangladeshi	4089 (0.65)	*	*
Other Asian	8756 (1.40)	*	*
Caribbean	5995 (0.96)	*	*
Black African	13847 (2.21)	*	*
Chinese	3522 (0.56)	*	*
Other ethnic group	17080 (2.73)	*	*
no CKD	597837 (95.40)	101 (58.05)	126 (70.39)
CKD3	25997 (4.15)	60 (34.48)	48 (26.82)
CKD4	1445 (0.23)	9 (5.17)	*
CKD5 only	701 (0.11)	*	*
CKD5 with dialysis	214 (0.03)	*	*
CKD5 with transplant	462 (0.07)	*	*
No chemotherapy in last 12	622837 (99.39)	170 (97.70)	173 (96.65)
Chemotherapy group A	1388 (0.22)	*	*
Chemotherapy group B	2250 (0.36)	*	*
Chemotherapy group C	181 (0.03)	*	*

No type2 diabetes	573141 (91.46)	126 (72.41)	129 (72.07)
Type 2 HBA<=59 mmol/mol (<=7.5%)	35001 (5.59)	34 (19.54)	30 (16.76)
Type2 HBA1C >59 (>7.5%)	18302 (2.92)	14 (8.05)	20 (11.17)
Type 2 HBA1C not recorded	212 (0.03)	*	*
Blood cancer	3675 (0.59)	*	*
Bone marrow or solid organ transplant	154 (0.02)	*	*
Respiratory cancer	1420 (0.23)	*	*
Radiotherapy in last 6 months	869 (0.14)	*	*
Down's syndrome	361 (0.06)	*	*
COPD	19355 (3.09)	28 (16.09)	19 (10.61)
Coronary heart disease	28061 (4.48)	46 (26.44)	32 (17.88)
Stroke	15750 (2.51)	24 (13.79)	23 (12.85)
Atrial fibrillation	17327 (2.76)	39 (22.41)	24 (13.41)
Heart Failure	8051 (1.28)	22 (12.64)	9 (5.03)
Venous thromboembolism	12950 (2.07)	16 (9.20)	15 (8.38)
Peripheral vascular disease	5881 (0.94)	16 (9.20)	*
Dementia	5156 (0.82)	33 (18.97)	22 (12.29)
Parkinson’s Disease	1633 (0.26)	*	5 (2.79)
Epilepsy	9968 (1.59)	*	*
Rare neurological conditions	2361 (0.38)	*	*
Liver cirrhosis	1448 (0.23)	*	*
Sickle cell disease	215 (0.03)	*	*
HIV or AIDS	1720 (0.27)	*	*
Severe combined immunodeficiency	307 (0.05)	*	*

*values<5 suppressed

Supplementary Table 2 Performance of the QCOVID3 risk prediction models in the validation cohort for COVID-19 death and COVID-19 hospital admission by ageband.

Ageband	Statistic	COVID-19 death	COVID-19 admission
< 60 years	C statistic*	98.4	88.2
	R^2	82.5 (71.7 to 93.3)	62.4 (50.7 to 74.1)
	Royston's D Statistic	4.44 (2.78 to 6.11)	2.64 (1.98 to 3.29)
60-69 years	C statistic*	76.7	84.1
	R^2	61.5 (44 to 78.9)	63.8 (50.5 to 77.1)
	Royston's D Statistic	2.58 (1.63 to 3.54)	2.72 (1.94 to 3.5)
70-79 years	C statistic*	81.9	70.8
	R^2	64 (53.6 to 74.5)	51.7 (39.8 to 63.6)
	Royston's D Statistic	2.73 (2.11 to 3.35)	2.12 (1.61 to 2.62)
80+ years	C statistic*	78.4	70.5
	R^2	50.7 (42.6 to 58.8)	39.9 (30.3 to 49.5)
	Royston's D Statistic	2.08 (1.74 to 2.41)	1.67 (1.33 to 2)

*unable to calculate confidence interval.

Supplementary Table 3 Performance of the QCOVID2 risk models in the validation cohort for COVID-19 death and hospital admission

	QCOVID2 females	QCOVID1 females	QCOVID2 males	QCOVID1 males
**FULL MODEL**				
**COVID-19_death**				
Harrell's C	.87 (.856 to .884)	.906 (.895 to .918)	.869 (.857 to .881)	.905 (.895 to .915)
R2	71.1 (69.8 to 72.4)	71 (69.7 to 72.3)	70.4 (69.2 to 71.6)	70 (68.7 to 71.2)
Royston's D Statistic	3.21 (3.11 to 3.31)	3.2 (3.1 to 3.3)	3.15 (3.06 to 3.24)	3.13 (3.03 to 3.22)
**COVID-19 admission**				
Harrell's C	.794 (.786 to .803)	.786 (.777 to .794)	.828 (.821 to .835)	.822 (.815 to .829)
R2	46.3 (44.8 to 47.8)	44.9 (43.3 to 46.4)	51.8 (50.5 to 53.1)	50.7 (49.4 to 52)
Royston's D Statistic	1.9 (1.84 to 1.96)	1.85 (1.79 to 1.9)	2.12 (2.07 to 2.18)	2.07 (2.02 to 2.13)
**RESTRICTED MODEL**				
**COVID-19_death**				
Harrell's C	.954 (.949 to .959)	NA	.947 (.942 to .952)	NA
R2	70.6 (69.1 to 72)	NA	70.1 (68.7 to 71.5)	NA
Royston's D Statistic	3.17 (3.06 to 3.28)	NA	3.13 (3.03 to 3.24)	NA
**COVID-19 admission**				
Harrell's C	.822 (.814 to .831)	NA	.848 (.842 to .854)	NA
R2	50.7 (49.2 to 52.2)	NA	54.6 (53.3 to 55.9)	NA
Royston's D Statistic	2.07 (2.01 to 2.14)	NA	2.24 (2.18 to 2.3)	NA

Supplementary Figure 1 Adjusted hazard ratio (95% CI) by age for risk of COVID-19 mortality

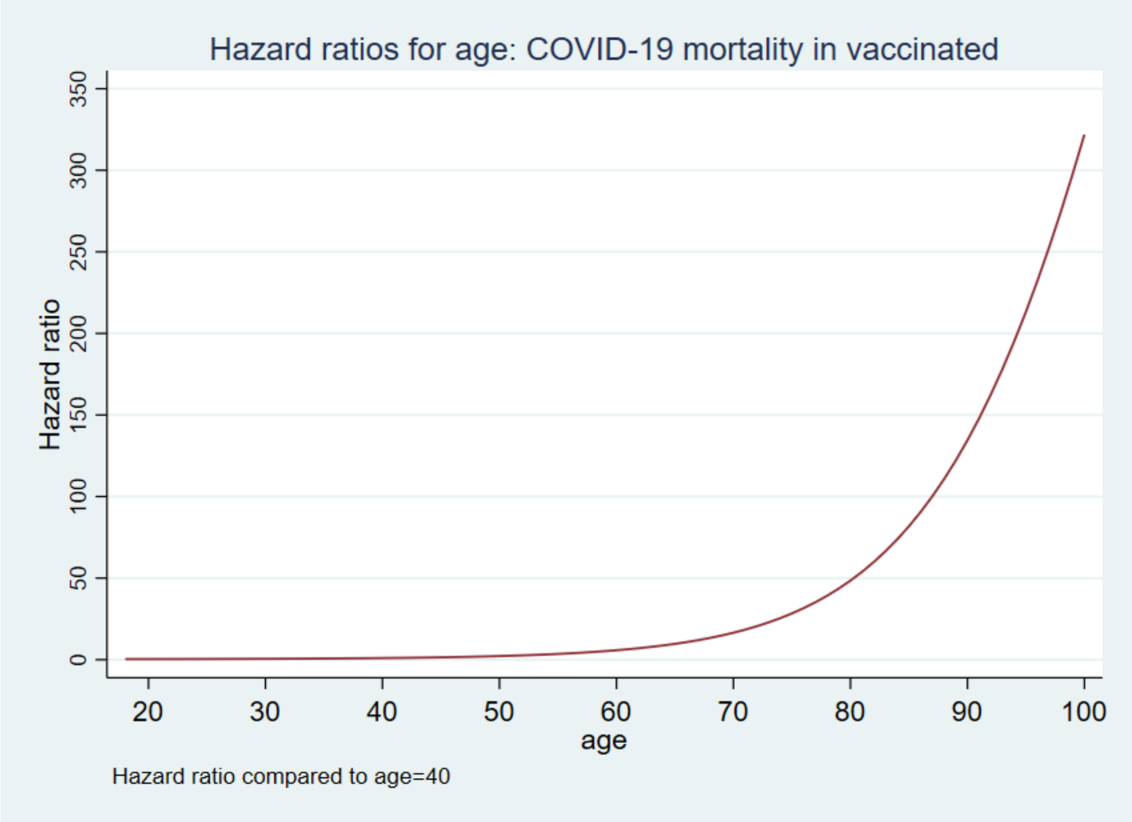

Supplementary Figure 2 Adjusted hazard ratio (95% CI) by BMI for COVID-19 mortality

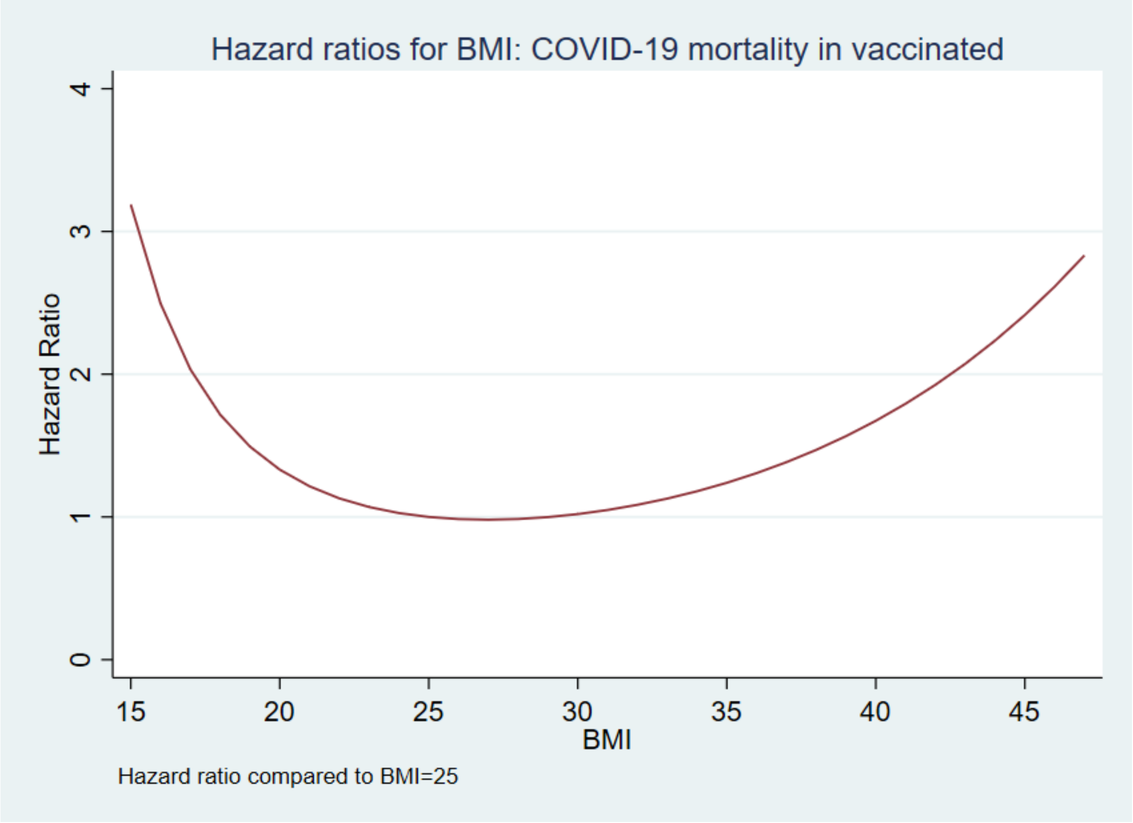

Supplementary Figure 3 Adjusted hazard ratio (95% CI) by prevailing SARS-CoV-2 infection rates for COVID-19 mortality

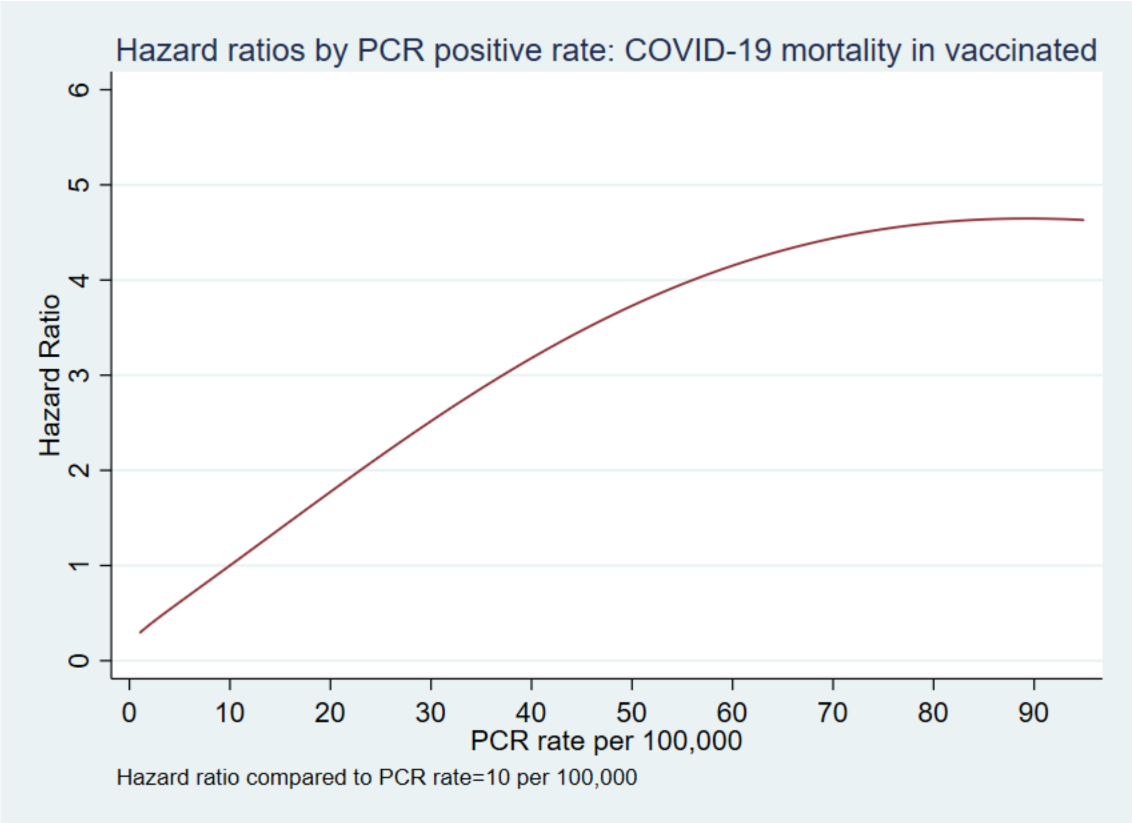

Supplementary Figure 4 QCOVID3: Adjusted cause specific hazard ratios for non-COVID-19 death after vaccination, mutually adjusted and also adjusted for fractional polynomial terms for age, BMI, vaccination dose and background infection rate in at the time of vaccination

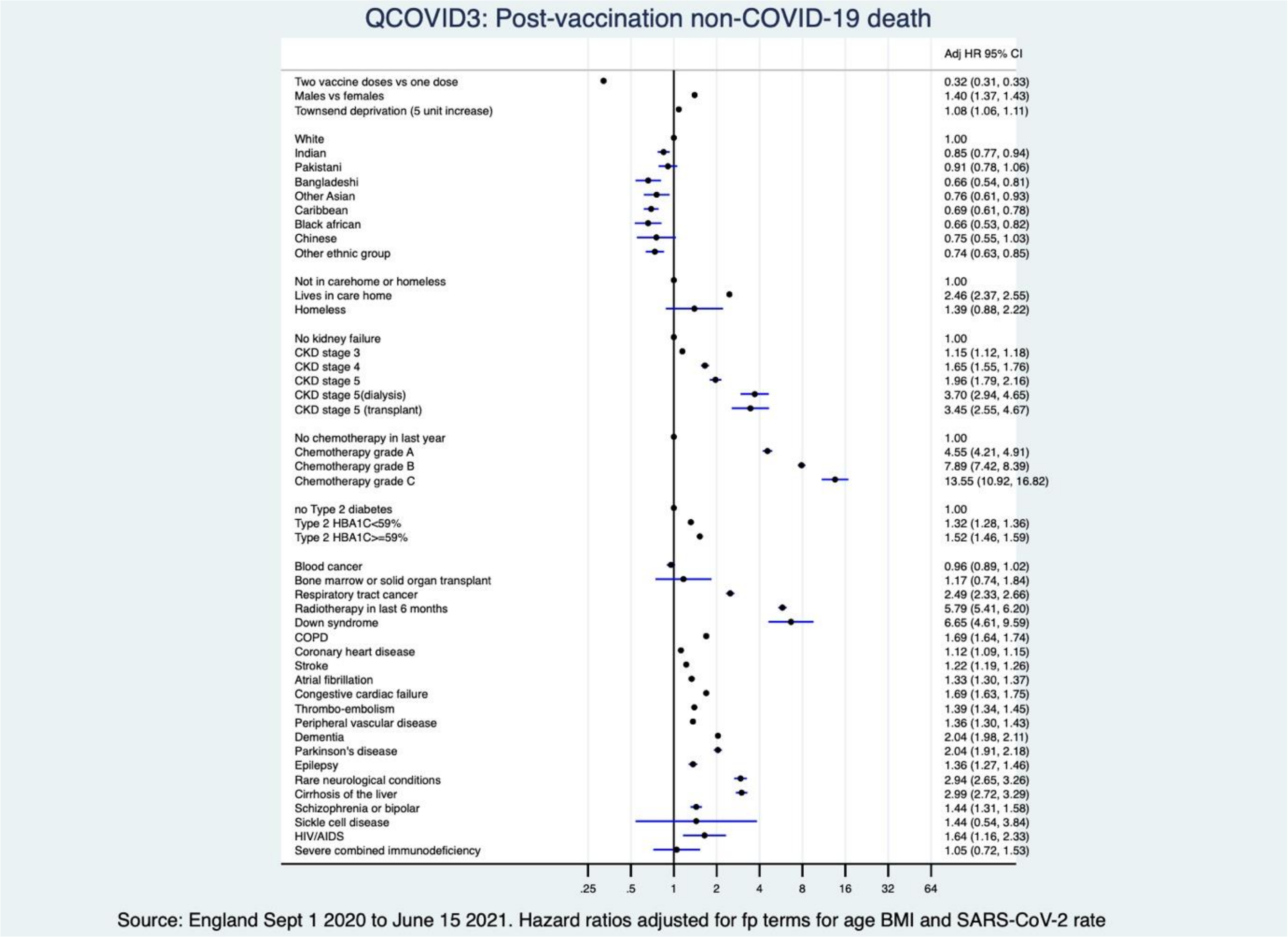

Supplementary Figure 5 QCOVID3: Adjusted cause specific hazard ratios for all-cause mortality after vaccination, mutually adjusted and also adjusted for fractional polynomial terms for age, BMI, vaccination dose and background infection rate in at the time of vaccination

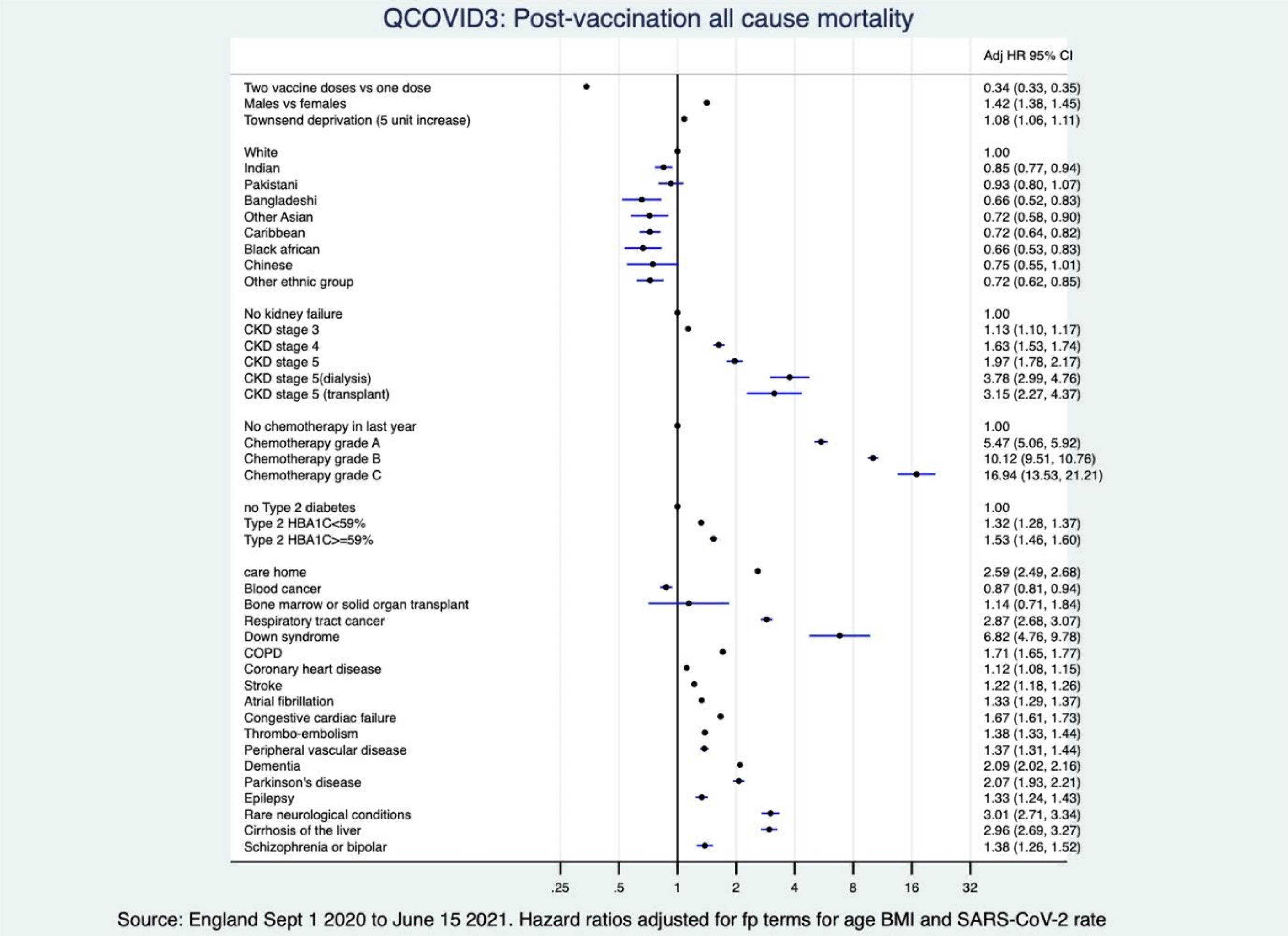

Supplementary Figure 6 QCOVID3. Adjusted cause specific hazard ratios for COVID-19 death after vaccination, mutually adjusted and also adjusted for fractional polynomial terms for age, BMI, background infection rate and vaccination dose among those with a SARS-CoV-2 positive test.

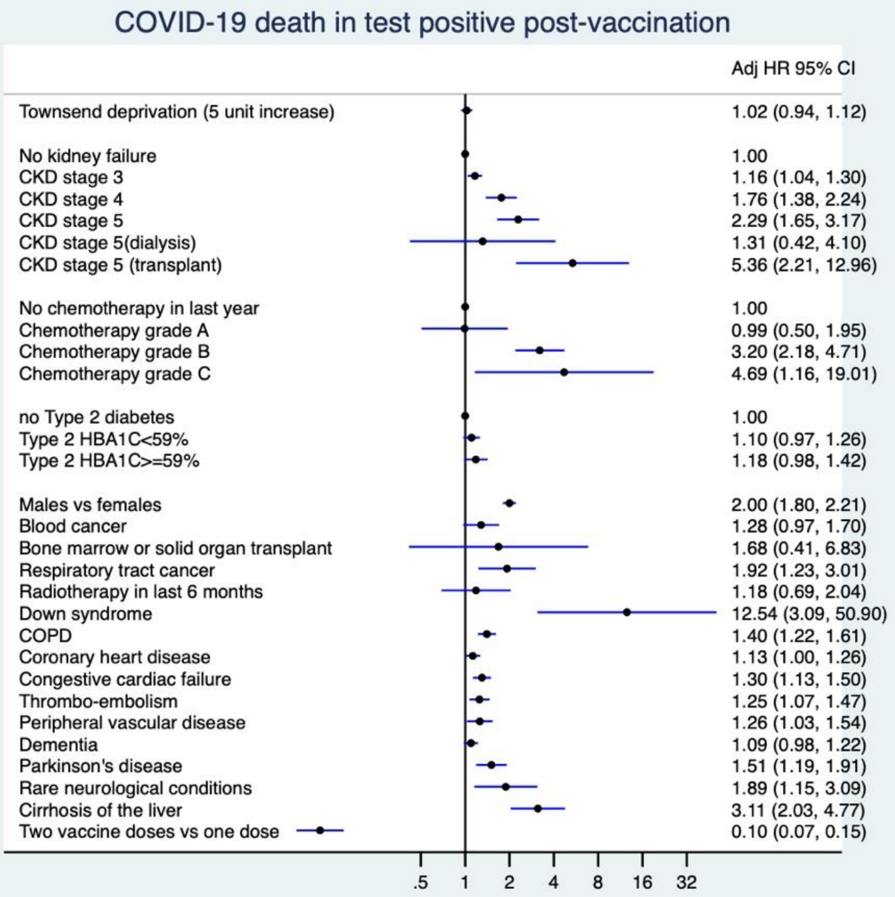

Figure 1 Adjusted HR (95%CI) for COVID-19 Outcomes following vaccination in England between Sept 1 2020 to April 24 2021
Models also adjusted for fp terms for age SARS-CoV-2 rate BMI; interactions between age and BMI and age and diabetes

Supplementary Figure 7 QCOVID2 Adjusted cause specific hazard ratios (95% CI) for risk of COVID-19 death in unvaccinated men and women during the second wave in England, mutually adjusted and also adjusted for fractional polynomial terms for age and BMI.

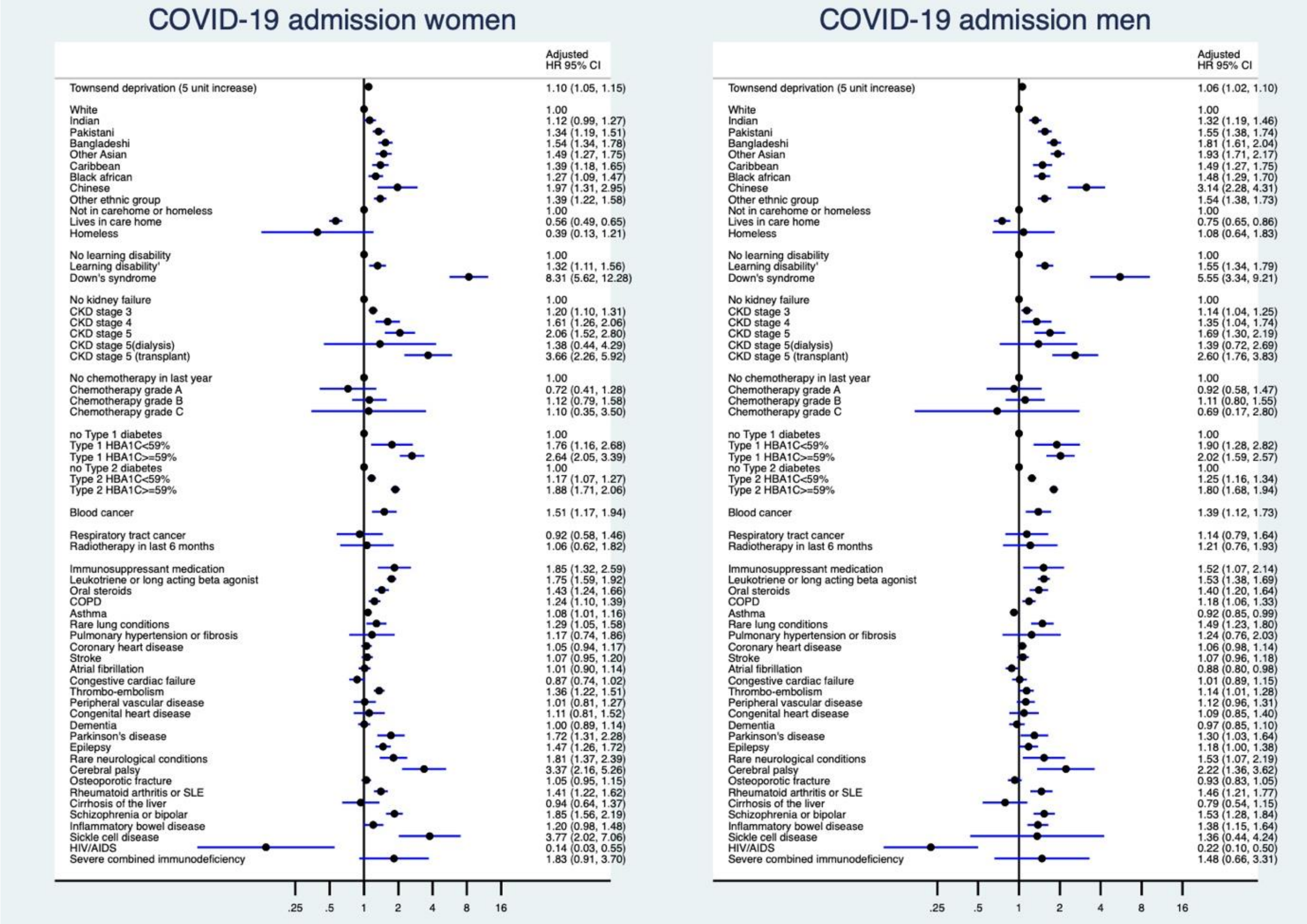

Notes: England Sept 1 2020 to May 31 2021. restricted QCOVID2 model hazard ratios adjusted for fp terms for age and BMI and interaction terms between age and BMI; age and type2 diabetes

Supplementary Figure 8 QCOVID2 Adjusted cause specific hazard ratios (95% CI) for risk of COVID-19 admission in unvaccinated men and women during the second wave in England, mutually adjusted and also adjusted for fractional polynomial terms for age and BMI.

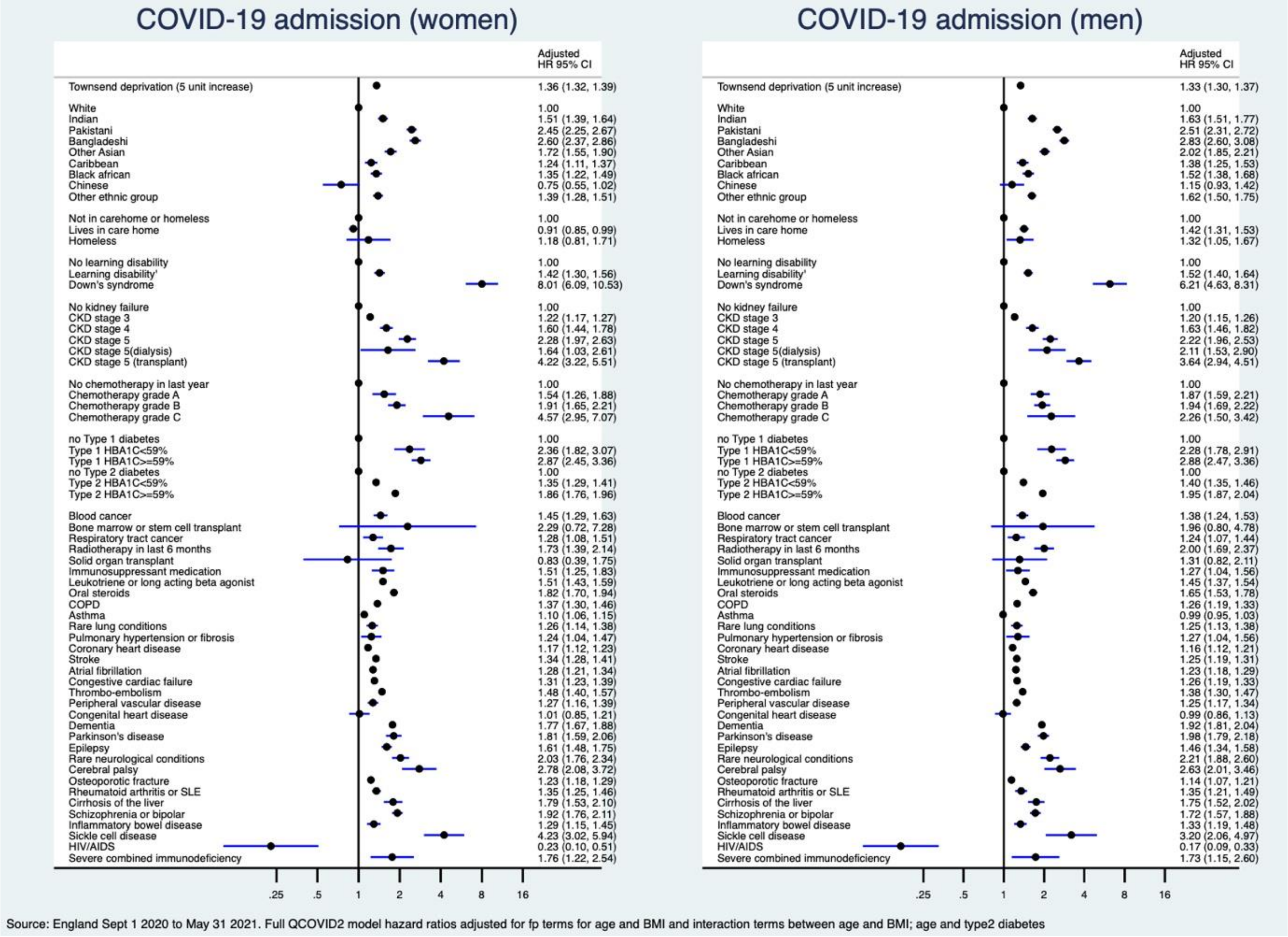

Supplementary Figure 9 QCOVID2 Adjusted cause specific hazard ratios (95% CI) for risk of COVID-19 death in unvaccinated men and women during the second wave in England, mutually adjusted and also adjusted for fractional polynomial terms for age and BMI among those with a SARS-CoV-2 positive test.

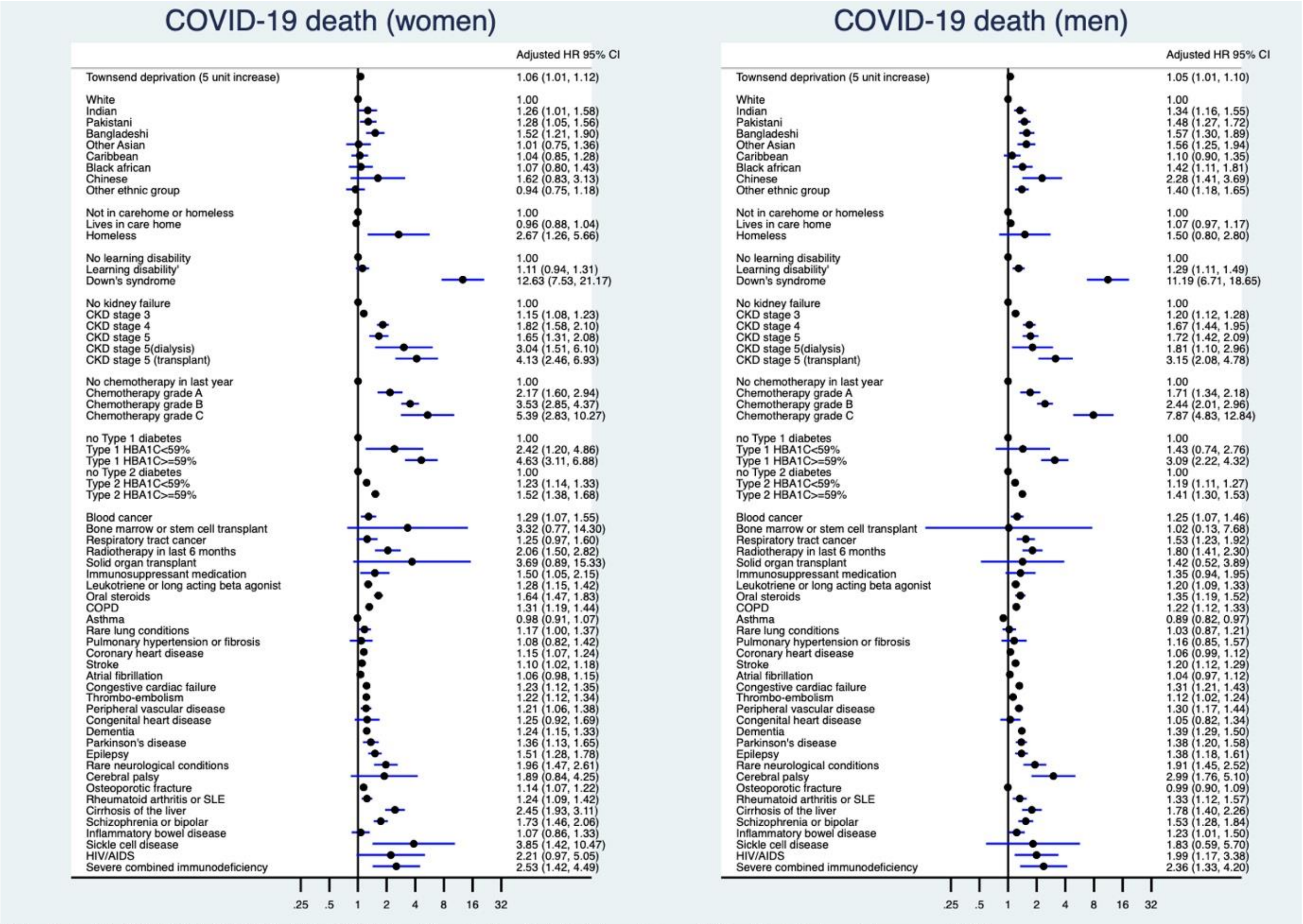

Notes: England Sept 1 2020 to May 31 2021. Restricted QCOVID2 model hazard ratios adjusted for fp terms for age and BMI and interaction terms between age and BMI

Supplementary Figure 10 QCOVID2 Adjusted cause specific hazard ratios (95% CI) for risk of COVID-19 admission in unvaccinated men and women during the second waves in England, mutually adjusted and also adjusted for fractional polynomial terms for age and BMI among those with a SARS-CoV-2 positive test.

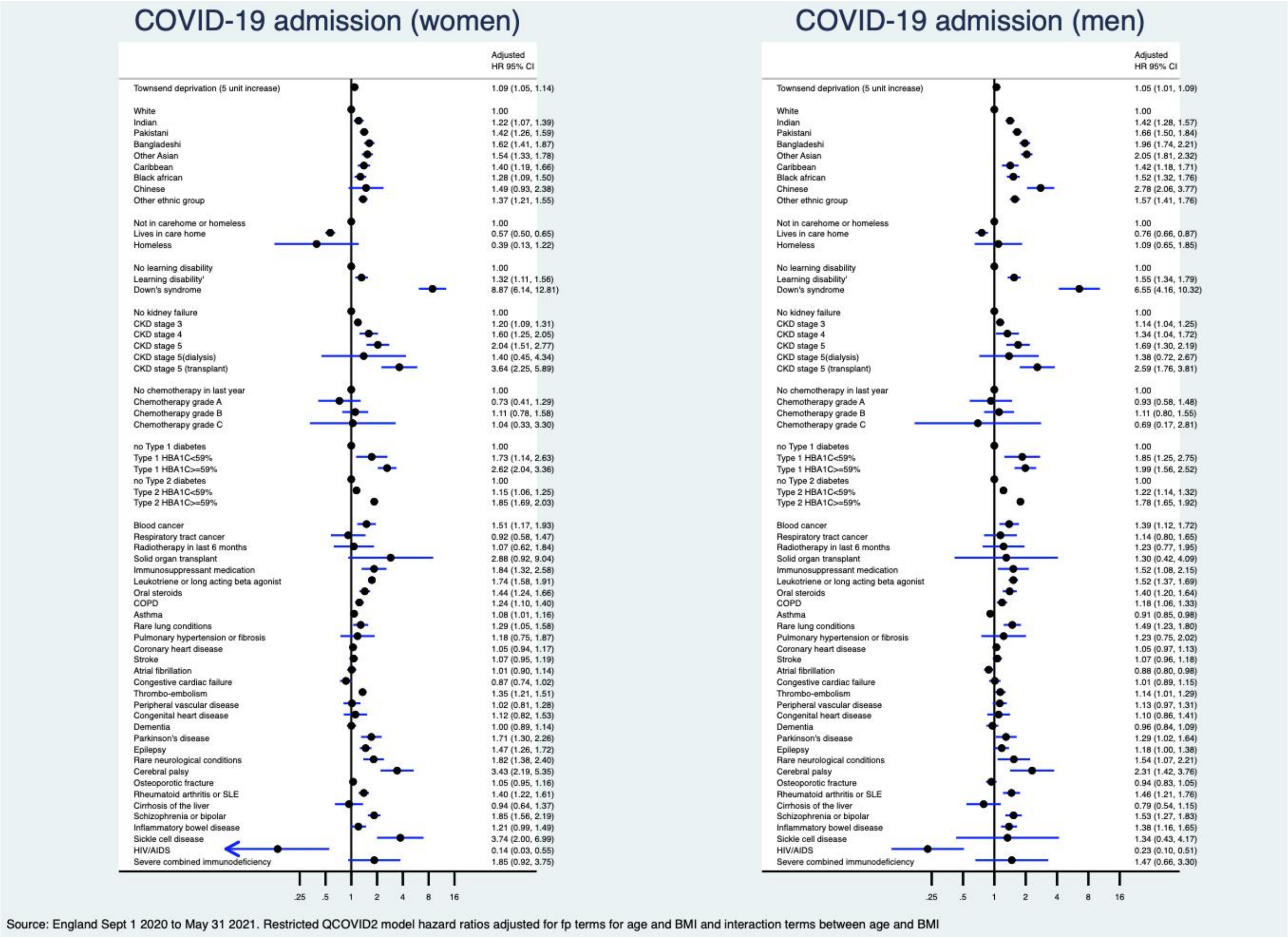

Supplement: Supplementary file 1 — Web appendix: Supplementary material [file hipj067627.ww.pdf]
